# Laparoscopic Resection for Rectal Cancer in a Centenarian: A Case Report Highlighting the Importance of Functional Independence over Chronological Age

**DOI:** 10.70352/scrj.cr.25-0653

**Published:** 2026-02-26

**Authors:** Asahi Tokinaga, Fuminori Teraishi, Naoki Onoda, Koki Omoto, Ryusei Takahashi, Hiroki Okabayashi, Masashi Utsumi, Hideaki Miyaso, Shinya Otsuka, Masaru Inagaki

**Affiliations:** Department of Surgery, NHO Fukuyama Medical Center, Fukuyama, Hiroshima, Japan

**Keywords:** centenarian, functional independence, geriatric assessment, laparoscopic surgery, rectal cancer

## Abstract

**INTRODUCTION:**

Laparoscopic resection for rectal cancer in a centenarian is extraordinarily rare, with only a few cases of elective curative surgery reported worldwide. As the global population ages, the number of centenarians with malignancies is increasing; however, surgical intervention in this age group remains controversial due to frailty and limited physiological reserve.

**CASE PRESENTATION:**

We present the case of a 100-year-old female with Stage IIIB rectal cancer who successfully underwent elective laparoscopic low anterior resection. Comprehensive preoperative geriatric assessments—comprising the Geriatric-8 (G8), instrumental activities of daily living (IADL), and EuroQol-5 dimensions (EQ-5D and EQ-VAS)—demonstrated excellent functional independence and physiological fitness. Despite severe intra-abdominal adhesions from a prior laparotomy, meticulous laparoscopic adhesiolysis and tumor-specific mesorectal excision were achieved without complications. Twelve lymph nodes were retrieved, with one positive node, and all resection margins were negative. The patient recovered uneventfully, retained postoperative independence, and was discharged on POD 12.

**CONCLUSIONS:**

This case highlights that functional and biological fitness—rather than chronological age—should guide surgical decision-making in the oldest-old population. It also underscores the feasibility and safety of minimally invasive curative surgery for selected centenarians when supported by detailed geriatric evaluation and multidisciplinary planning.

## Abbreviations

CGA
: comprehensive geriatric assessment
EQ-5D
: EuroQol-5 dimensions
EQ-VAS
: EuroQol-5 visual analog scale
G8
: Geriatric-8
IADL
: instrumental activities of daily living

## INTRODUCTION

The global increase in life expectancy has led to a growing number of centenarians, with cancer now recognized as a significant cause of morbidity and mortality, even among the oldest-old population. Recent epidemiological data suggest that although the overall cancer burden declines after the age of 85 years, certain malignancies such as colorectal, breast, and prostate cancers remain relatively prevalent in centenarians.^1)^ Among these, colorectal cancer is particularly noteworthy because of its increasing incidence and potential for curative treatment even at an advanced age.^2)^

However, surgical intervention in this age group remains controversial primarily because of concerns regarding frailty, limited physiological reserves, and increased perioperative risk.^3)^ Historically, extreme old age has often been considered a contraindication to major surgery, including oncologic resection, and many centenarians have been managed with nonoperative or palliative approaches. Nevertheless, emerging reports have highlighted that select super-elderly individuals, particularly those with preserved ADL and minimal comorbidities, may tolerate surgery remarkably well.^2,4)^

Herein, we report the rare case of a 100-year-old female with rectal cancer who successfully underwent curative laparoscopic low-anterior resection. Preoperative assessments, including the G8 screening tool, IADL, EQ-5D, and EQ-VAS confirmed sufficient functional independence and physiological resilience to justify a surgical approach. To our knowledge, reports of laparoscopic curative resection for rectal cancer in centenarian patients are exceedingly scarce. A recent literature search revealed only isolated cases of colorectal surgery in individuals aged ≥100 years, most of which were palliative or emergent rather than elective procedures. The present case therefore represents one of the few documented instances of elective laparoscopic low anterior resection with curative intent in a centenarian, underscoring its rarity and clinical significance. This case underscores the potential of individualized treatment strategies based on functional status rather than chronological age alone.

## CASE PRESENTATION

**Fig. 1:**
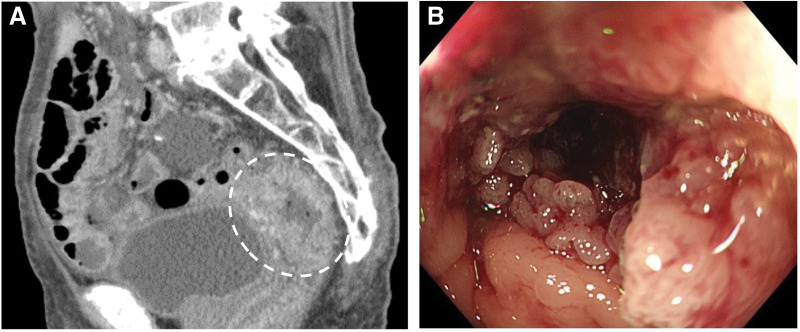
Preoperative imaging studies. **(A)** Contrast-enhanced pelvic CT shows a 40-mm segment of wall thickening with enhancement in the rectosigmoid region. **(B)** Colonoscopic examination reveals a type 2 tumor occupying approximately three-fourths of the lumen circumference, located 20 cm from the anal verge.

A 100-year-old female presented at the emergency department with recurrent abdominal pain. Her medical history included small bowel obstruction treated surgically 40 years prior with resection of 80 cm of the intestine. Additional comorbidities included hypertension and cataracts. Initial evaluation suggested adhesive small bowel obstruction; however, abdominal CT incidentally revealed a 4-cm segment of irregular rectal wall thickening extending from the Ra to the Rs with suspected serosal invasion (cT4a), one enlarged perirectal lymph node (N1a). Subsequent colonoscopy revealed a circumferential type 2 tumor located 20 cm from the anal verge that partially obstructed the lumen (**Fig. 1**). Biopsy confirmed a moderately differentiated tubular adenocarcinoma. A clinical diagnosis of Stage IIIB rectal cancer (cT4aN1aM0) was established.

The patient was notably independent in daily life, living alone, and managing all ADL and IADL without assistance. Her height and weight were 139.0 cm and 37.8 kg, respectively. Upon examination, the patient developed mild abdominal distension and tenderness. Laboratory data revealed mild anemia (hemoglobin, 10.3 g/dL), severe renal impairment (creatinine clearance, 18.4 mL/min), and a serum albumin concentration of 3.5 g/dL. Carcinoembryonic antigen level was elevated (9.25 ng/mL).

Geriatric assessments performed by the attending surgeon revealed a G8 score of 11, IADL score of 8, EQ-5D index of 1.000, and EQ-VAS score of 80, reflecting preserved functional and cognitive reserves (**Table 1**). Echocardiography revealed a left ventricular ejection fraction of 69%, with no significant valvular disease. The pulmonary function was sufficient for general anesthesia.

**Table 1:** Preoperative, 1-month postoperative, and 3-months postoperative comprehensive geriatric assessments

	Pre-operation	1-month after operation	3-months after operation
G8 score	11	10	13
IADL	8	5	5
EQ5D	1.000	0.665	0.781
EQ5D-VAS	80	70	70

EQ5D, EuroQol-5 dimensions; EQ-VAS, EuroQol-5 visual analog scale; G8, Geriatric 8; IADL, instrumental activities of daily living

**Fig. 2:**
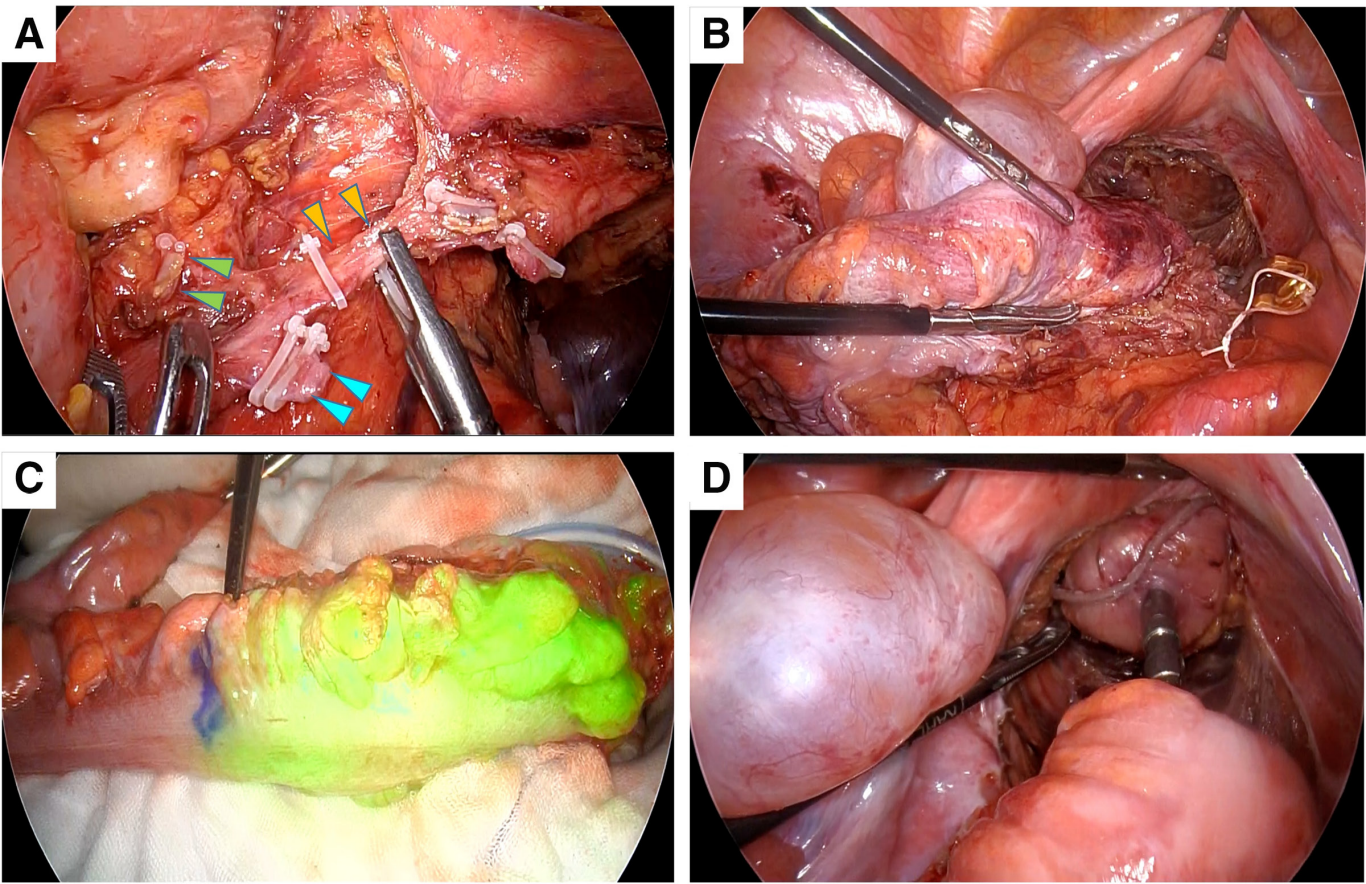
Intraoperative photographs. A medial approach was employed to achieve central vascular ligation, during which the superior rectal artery stump (blue arrowheads), inferior mesenteric vein stump (green arrowheads), and sigmoid artery (orange arrowheads) were clearly identified **(A)**. The right upper panel demonstrates tumor-specific mesorectal excision **(B)**, the left lower panel illustrates indocyanine green-based assessment of proximal sigmoid colon perfusion **(C)**, and the right lower panel depicts reconstruction performed using the double-stapling technique **(D)**.

**Fig. 3:**
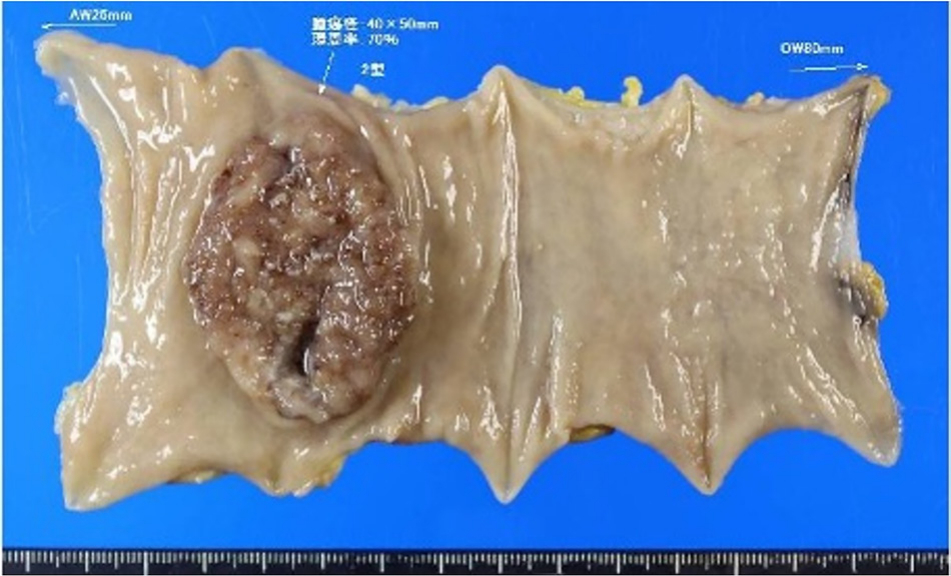
In the resected specimen, a type 2 tumor measuring 4 × 5 cm and involving approximately 70% of the rectal circumference was identified.

Given her good functional status and absence of contraindications, an elective laparoscopic low-anterior resection with D2 lymphadenectomy was scheduled. Dense intra-abdominal adhesions were encountered because of a prior surgery. For adhesions adjacent to the intestine, sharp dissection with scissors was performed to minimize the risk of serosal injury. Extensive adhesions between the abdominal wall and the omentum were managed using blunt dissection combined with adhesiolysis using an electrosurgical device. In particular, dense adhesions involving the small bowel loops and the previous anastomotic site required meticulous, layer-by-layer dissection to ensure a safe operative field. This step significantly contributed to the prolonged operative time (5 hours) and highlights the technical challenges of laparoscopic surgery in patients with a history of prior open abdominal surgery. The superior rectal and sigmoid arteries were ligated, and tumor-specific mesorectal excision was performed. Intraoperative indocyanine green fluorescence imaging confirmed sufficient anastomotic perfusion. A double-stapling technique was used in this study (**Fig. 2**). No intraoperative complications occurred. The operative time was 5 hours, with an estimated blood loss of 100 mL. The excised specimen showed tumor-specific mesorectal excision, the distal margin was secured, and the circumferential margin showed no exposed cancer (**Fig. 3**). Histopathological examination revealed a moderately differentiated tubular adenocarcinoma without evidence of lymphovascular or perineural invasion. Twelve lymph nodes were examined, of which one showed metastatic involvement (1/12). The circumferential and distal resection margins were free of tumor infiltration. Accordingly, the pathological diagnosis was T3N1aM0, corresponding to Stage IIIB disease.

Regarding perioperative rehabilitation, physical therapists provided preoperative respiratory training and exercise-based rehabilitation with an emphasis on ambulation. Postoperatively, bed-based exercises were initiated on POD 1, followed by mobilization on POD 2, with a gradual progression to walking training. The patient resumed drinking on POD 1, transitioned to a soft diet on POD 4, and was discharged on POD 12 without complications. At 1 and 3 months after surgery, comprehensive geriatric assessments were conducted during outpatient visits. The patient’s G8 scores were 10 and 13, IADL scores were 5 and 5, EQ-5D indices were 0.665 and 0.781, and EQ-VAS scores were 70 and 70, respectively. Although the IADL and EQ-5D index scores showed a slight decline compared with preoperative levels, the G8 score improved beyond the preoperative value by the 3-month assessment (**Table 1**). At the 3-month postoperative follow-up for IADL, three items—*shopping*, *meal preparation*, and *transportation*—were each rated as 0 points (data not shown). After thorough discussion of the postoperative treatment plan with the patient and her caregiver, they declined adjuvant chemotherapy; therefore, the patient was managed with careful observation alone.

## DISCUSSION

This case highlights successful curative laparoscopic resection of rectal cancer in a 100-year-old patient, emphasizing the preservation of functional independence and feasibility of minimally invasive surgery, even in individuals of extreme age. Although centenarians are underrepresented in the colorectal surgical literature, our report contributes to the growing evidence that with appropriate selection and evaluation, curative oncologic surgery can be safely performed in this population. While cancers such as breast, prostate, and skin malignancies have occasionally been reported in centenarians, colorectal cancer requiring curative resection remains exceptionally uncommon in this age group. Literature reporting resection for colorectal cancer in individuals over 100 year old is limited to sporadic case reports. Thus, this report adds valuable evidence regarding the feasibility of minimally invasive oncologic resection in the oldest-old population.

Previous reports on colorectal surgery in centenarians have largely described procedures undertaken in palliative or emergent settings, such as bowel obstruction.^4)^ Elective curative resections are rare. Sotiropoulos et al. described a sigmoid colectomy in a 100-year-old male, though without the benefit of a detailed preoperative functional assessment or laparoscopic approach.^5)^ By contrast, our patient underwent elective laparoscopic low anterior resection after a comprehensive geriatric evaluation including G8, IADL, and EQ-5D assessments. These tools demonstrate preserved physical and cognitive function and provide a sound basis for curative surgery.

Chronological age alone should not be viewed as a contraindication for surgery. However, advanced age is associated with increased perioperative risk, frailty, and comorbidities.^6,7)^ With careful selection, outcomes in nonagenarians and even centenarians may approximate those observed in younger patients.^6)^ Sudlow et al. found acceptable surgical outcomes in nonagenarians when baseline independence and minimal comorbidities were present.^6)^ Our patient exhibited excellent functional status, no major organ dysfunction, and favorable G8 screening results, all of which supported the decision to undergo surgery.

Anesthetic risk is a well-recognized concern in this population. Irwin et al. noted that age-related declines in cardiovascular, pulmonary, and renal reserves necessitate careful planning.^7)^ Nonetheless, the use of minimally invasive surgery and multimodal perioperative strategies can mitigate stress responses and enhance recovery. In this case, general anesthesia was safely administered and the patient recovered without complications, underscoring the value of multidisciplinary planning.

The laparoscopic approach provides significant benefits to older patients, including reduced postoperative pain, faster recovery, and shorter hospitalization. While data specific to centenarians are limited, studies in nonagenarian cohorts confirm its safety and efficacy.^5,6)^ Our patient resumed oral intake early, had no postoperative complications, and was discharged on day 12, suggesting that laparoscopy contributed to the favorable outcomes.

The distinction between biological and chronological ages is particularly relevant in centenarian care. Recent studies have suggested that centenarians may exhibit genetic traits associated with longevity, including reduced pathogenic variant burden and greater genomic stability.^8)^ Torres et al. have reported a lower prevalence of age-related disease-associated variants and higher polygenic longevity scores in long-lived individuals.^8)^ Although genetic testing was not conducted in our case, the patient’s clinical robustness may reflect underlying biological resilience.

The necessity of lymph node dissection for colorectal cancer in the oldest-old patients remains controversial. Although guideline-based treatment is generally recommended for patients who are fit for surgery, we recently reported that, while D3 lymph node dissection can be safely performed in patients aged over 90 years with colorectal cancer, its impact on improving prognosis appears to be limited.^9)^ In the present case, a preoperative multidisciplinary conference concluded that D2 dissection would be sufficient, balancing surgical safety with curative intent. Psychosocial factors also play critical roles in surgical candidacy and recovery. Aliberti et al. emphasized the importance of lifestyle, diet, and social engagement in the well-being of centenarians.^10)^ The patient’s independent living status likely contributed positively to her overall resilience. In this case, at the 3-month postoperative follow-up, 3 IADL items—*shopping*, *meal preparation*, and *transportation*—were each scored as 0 points, resulting in an overall IADL score of 5. This outcome was largely attributable to a change in living circumstances, from living alone preoperatively to residing with her daughter’s family after discharge. Consequently, daily shopping and meal preparation are currently performed by family members, and opportunities for independent transportation have been limited during the early postoperative period.

Emerging tools, such as the GERIATRIC risk stratification system, aim to predict outcomes in super-elderly surgical candidates by incorporating frailty indices and physiological data.^11)^ Although chronological age is often regarded as a major determinant in surgical decision-making, CGA should be understood as a framework for evaluating physiological reserve rather than as an absolute criterion for treatment selection. In the present case, favorable CGA findings—particularly preserved functional independence, intact cognitive function, acceptable nutritional status, and adequate social support—supported the feasibility of curative-intent surgery. Consistent with previous reports, CGA was used not as a stand-alone determinant, but as a structured tool to identify vulnerabilities and strengths that may influence perioperative risk and postoperative recovery.^12,13)^ Treatment decisions were therefore made by integrating CGA results with oncological factors such as clinical stage and resectability, anticipated surgical invasiveness, and patient preferences.

Conversely, even in patients of advanced age, a non-surgical or palliative approach may be more appropriate when CGA reveals severe functional dependency, advanced cognitive impairment, frailty with limited reversibility, or insufficient social support, particularly when these factors outweigh potential oncological benefit. At the same time, CGA has inherent limitations: while it provides a systematic assessment of vulnerability, it does not fully capture surgical complexity or individual values regarding QOL. Therefore, CGA should be viewed as an essential component—rather than a definitive determinant—within a multidisciplinary and individualized decision-making process.

This case is, to our knowledge, among the first detailed reports of laparoscopic low anterior resection for rectal cancer in a centenarian with documented pre- and postoperative geriatric functional assessments. The integration of G8, IADL, and EQ-5D evaluation into perioperative decision-making provides a novel perspective on surgical candidacy in the extreme elderly, emphasizing the paradigm shift toward biological and functional rather than chronological assessment of operative risk.

## CONCLUSIONS

In conclusion, this case report demonstrates that curative laparoscopic surgery is feasible in centenarians when comprehensive functional and physiological assessments are favorable. Age alone should not dictate the treatment strategy. Further prospective studies and a wider use of geriatric assessment tools will aid in refining surgical indications for the oldest-old population.
